# P38MAP kinase, but not phosphoinositol-3 kinase, signal downstream of glutamine-mediated fibronectin-integrin signaling after intestinal injury

**DOI:** 10.1186/1475-2891-12-88

**Published:** 2013-06-21

**Authors:** Stefanie Niederlechner, Christine Baird, Paul E Wischmeyer

**Affiliations:** 1 Department of Anesthesiology, University of Colorado, 12700 E 19th Ave, P15-Box 8602, Colorado, Aurora, 80045, USA

**Keywords:** Apoptosis, HSP70, ERK1/2, p38MAPK, GRGDSP

## Abstract

**Background:**

Glutamine appears to mediate protection against gut injury via multiple pathways. These include fibronectin-integrin, PI3-K/MAPK pathways, and activation of heat shock protein (HSP) response. We hypothesize there may be a relationship between these pathways mediating glutamine’s protection in intestinal epithelial-6 cells after heat stress. We assessed whether p38MAPK and PI3-K/Akt signaling are involved in glutamine’s cytoprotective mechanism and the role they play in glutamine-mediated protection in conjunction with fibronectin-integrin osmosignaling after hyperthermia.

**Methods:**

Intestinal epithelial cells were treated for 15 min with glutamine, with/without the fibronectin-integrin interaction inhibitor GRGDSP, inactive control peptide GRGESP, p38MAPK inhibitor SB203580, or PI3-K/Akt inhibitor LY294002 under basal (37°C) and stressed (43°C or 44°C) conditions. Cell survival was measured via MTS assay 24 h post-heat stress (44°C × 50 min). Total p38MAPK, [T(P)^180^/Y(P)^182^]p38MAPK, total Akt, [S(P)^473^]Akt, HSP70, FN, and caspase-3 levels were determined via Western blot after non-lethal HS (43°C × 50 min). Additionally, HSP70 levels were assessed via Western blot and ELISA.

**Results:**

We were able to show that GRGDSP and LY294002 attenuated glutamine’s protective effect. However, SB203580 increased cell survival after heat stress. LY294002 attenuated glutamine-mediated increases in fibronectin and in HSP70 expression after hyperthermia. GRGDSP increased glutamine-mediated attenuations in p38MAPK phosphorylation, but had no effect on glutamine-mediated augmentations in Akt phosphorylation.

**Conclusions:**

These data suggest that glutamine is protective after heat stress by activating PI3-K/Akt signaling preventing fibronectin-integrin expression and increasing HSP70 expression. Furthermore, dephosphorylation of p38MAPK after heat stress plays an important role in glutamine-mediated cellular protection. However, p38MAPK, but not PI3-K/Akt, signals downstream of glutamine-mediated fibronectin-integrin signaling after hyperthermia.

## Introduction

GLN is a crucial substrate for the small intestine and has a central role in numerous metabolic processes in the gut because of its preferential substrate status in this organ [1,2]. It serves not only as a precursor of protein, polyamines, glutathione, and nucleotide synthesis, and as a nitrogen carrier, but also as the primary metabolic fuel for enterocytes [2-5]. During illness and malnutrition, GLN-supplemented parenteral and enteral nutrition may become essential for the gut, because GLN reduces intestinal permeability, decreases bacterial translocation, enhances immune function, protects gut mucosa against injury, accelerates healing of the small intestine, and improves nitrogen balance in animal models of intestinal injury [6-10]. GLN has been studied extensively, however, its molecular mechanisms of action, especially the initial key steps, still remain unknown.

*In vitro* and *in vivo* studies have shown that GLN can provide protection by enhancing heat shock protein (HSP) expression [11,12]. HSPs are highly conserved proteins involved in the most basic mechanisms of cellular protection. HSP induction can cause ‘stress tolerance’ and provide protection from subsequent stress that would otherwise be lethal [13-15]. However, the pathway by which GLN induces HSP expression appears to be complex and multifaceted.

GLN is an osmotically acting amino acid, which is co-transported with sodium into the cell. This causes an influx of water and induces a ‘cell-swelling’ effect [16]. Osmotic changes are a major physical stress that all cells undergo. Thus, osmotic-linked cell signaling (osmosignaling) plays an essential role in the activation of specific survival genes [17]. A number of integral membrane proteins, including integrins have been assigned roles as upstream sensors of cell volume changes [17-19]. Integrins are a highly conserved family of heterodimeric adhesion molecules that connect the extracellular matrix (ECM) (e.g. fibronectin (FN)) to intracellular signaling proteins and the cytoskeleton [17,20]. This unique ability of integrins to regulate attachment of cells to ECM proteins is called “inside-out signaling” [21,22]. Ligand binding is transduced from the ECM to the cytosol by “outside-in signaling” [23]. Thus, integrins are able to transduce signals in both directions. FN-Integrin signaling can sense osmotic changes and was shown to be an essential key step in GLN’s protective mechanism via Erk1/2, HSF-1, and HSP70 signaling [24].

Further, MAPKs, as well as the phosphoinositol 3-kinase (PI3-K) pathways are crucial downstream survival signaling cascades from the membrane to the nucleus [25-27]. Recently, it could be shown that GLN is protective via ERK1/2 activation and p38MAPK dephoshorylation in IEC-6 cells after HS [28].

In this study we investigated whether p38MAPK and PI3-K/Akt signaling are involved in GLN’s cytoprotective mechanism and what role they play in GLN-mediated protection in conjunction with FN-Integrin osmosignaling after intestinal injury.

## Material and methods

All chemicals were purchased from Sigma-Aldrich (St. Louis, MO), unless otherwise specified.

### Cell culture

IEC-6 (ATCC, Manassas, VA) were grown in Dulbecco's modified Eagle's medium (DMEM), supplemented with 10% fetal bovine serum (FBS), 2 mM L-GLN, 10 ml/l of antibiotic solution containing penicillin G (10,000U/ml) and streptomycin (10,000 μg/ml) (Cellgro Mediatech), and 0.01 mg/ml insulin. Cultured cells were maintained in a humidified 37°C incubator with 5% CO_2_. GLN starvation was performed by depriving cells of GLN for 24 h in DMEM, supplemented with 10% FBS and 0.01 mg/ml insulin.

### Heat-stress injury

The model of heat stress injury in IEC-6 cells was used to mimic intestinal inflammation and injury, because it represents the most-widely accepted method for inducing a “stress protein response” or heat shock protein expression [29,30]. For cell viability, 96-well plates were submerged to a lethal heat stress in a 44°C Precision water bath Model 260 (Winchester, VI) for 50 min [31] and allowed to recover at 37°C for 24 h. For protein expression experiments, cells were subjected to a non-lethal heat stress at 43°C for 45 min [31] or remained for 45 min in the 37°C incubator (as control), followed by a 0 h or a 3 h recovery time.

### Protein extraction and Western blot analysis

Cells were seeded in 10 cm dishes and allowed to grow for 3d in full media. 24 h before the experiment, when the cells were approximately 80% confluent, the standard culture full media was replaced by GLN-free, serum containing DMEM with only 10% FBS and 0.01 mg/mL insulin for 24 h to standardize GLN content at the start of each experiment and to mimic the severe GLN depletion occurring in critical illness. Cells were, then, treated with/without 10 mM GLN for 15 min, with or without prior 1 h treatment with FN-Integrin inhibitor GRGDSP (50 μM, diluted in dH_2_0), inactive control peptide GRGESP (50 μM, diluted in dH_2_0) (Bachem, Torrance, CA), p38MAPK inhibitor SB203580 (10 μM and 30 μM, diluted in DMSO), or PI3-K inhibitor LY294002 (25 μM, diluted in DMSO) and subjected to HS. At the end of experimental treatment, medium was removed from the culture, and cells were immediately washed and harvested in ice-cold PBS. For total protein extraction cells were lysed at 4°C using 180 μl M-PER lysis buffer (Pierce, Rockford, IL) with inhibitor protease and phosphatase cocktail (Roche, Indianapolis, IN). Protein was determined with BCA protein assay (Pierce, Rockford, IL). 15 μg of each sample were added to a 4× treatment buffer (250 mM Tris/Cl, 8% SDS, 27.5% glycerol, 20% 2-mercaptoethanol, 0.1% bromphenol blue, pH 6.8), boiled for 3 min, and then loaded into a NuPAGE 4–12% Bis-Tris Gel (Invitrogen, Carlsbad, CA). Following electrophoresis, gels were equilibrated with transfer buffer (1.2 g Tris/Cl, 7.5 g glycine, 100 ml MeOH, 950 ml dH_2_O). Proteins were electrophoretically separated with a mini-gel system and transferred to polyvinylidine difluoride membranes (Millipore, Billerica, MA), using the biorad wet transfer system. Membranes were blocked with 5% nonfat milk in PBS-Tween or 5% bovine serum albumin (BSA) in PBS-Tween for 1 h at room temperature. Primary antibodies against total p38MAPK, [T(P)^180^/Y(P)^182^]p38MAPK, total Akt, [S(P)^473^]Akt, HSP70, caspase-3 (1:1,000) (Cell signaling, Danvers, MA), and FN were added to antibody buffer (blocking solution) and incubated overnight at 4°C. After washing three times with PBS-Tween over 30 min, secondary antibodies, peroxidase-conjugated goat anti-mouse or goat anti-rabbit IgG (Pierce), were applied at a 1:3,000 dilution for 1,5 h. Blots were washed three times with PBS-Tween over 30 min, incubated in commercial enhanced chemiluminescence reagents (Pierce), and exposed utilizing a UVP chemiluminescent darkroom system (UVP, Upland, CA). Densitometry was normalized against β-actin (1:50,000).

### HSP70 ELISA

HSP70 levels were also evaluated via HSP70 ELISA (StressGen, Belgium). IEC-6 cells were treated with the chemical inhibitor LY294002 (25 μM) as previously described, with or without subsequent GLN and heat stress injury, and allowed to recover for 3 h. Cells were collected, lysed, and assayed for total protein (as specified in the Western blot analysis section above). 10 μg of protein was used per well, and the ELISA was performed via manufacturer’s instructions.

### MTS cell viability assay

IEC-6 cells were seeded in 96 well plates (7,000 cells per well), and allowed to grow for 42 h in full media until 80% confluence. The cells were then cultured for 24 h in GLN-free, serum containing DMEM. After GLN-starvation for 24 h, cells were exposed to different concentrations of GLN (0 mM, 2 mM, 10 mM) for 15 min. 25 μM LY294002 or 30 μM SB203580 were used 1 h prior to GLN treatment to inhibit PI3-K and p38MAPK signaling. Cells were then subjected to lethal HS (as specified above). Cell viability was evaluated via a soluble tetrazolioum salt (MTS) assay (Promega, Madison, WI) as per manufacturer’s instructions 24 h later. Briefly, one part PMS was added to twenty parts MTS immediately before the solution was diluted 1:5 in phenol red-free DMEM and was added to IEC-6 cells. MTS was bioreduced by cells into a colored, soluble formazan product. Absorbance values were read after 3 h at 490 nm, using an ELISA plate reader (Thermo Electro Corporation, San Jose, CA); references included readings at 650 nm and no-cell blank wells. Higher absorbance values reflect greater cell viability. Every well (n = 4 per group in each experiment) was normalized to their individual non-HS controls, to account for possible differences in cell growth.

### Data analysis and statistics

All experiments were repeated at least 3 times with IEC-6 cells of different passage numbers [14-22]. Statistical analysis was validated with GraphPad Prism Analysis software. Conditions were compared by using one-way ANOVA, followed by Turkey’s post-hoc test, or student’s *t*-test where appropriate, and are expressed as means ± SEM (number of experiments). Differences were considered significant at *P* < .05.

## Results

### GLN is protective via PI3-K/Akt-HSP70 signaling after HS

**Figure 1:**
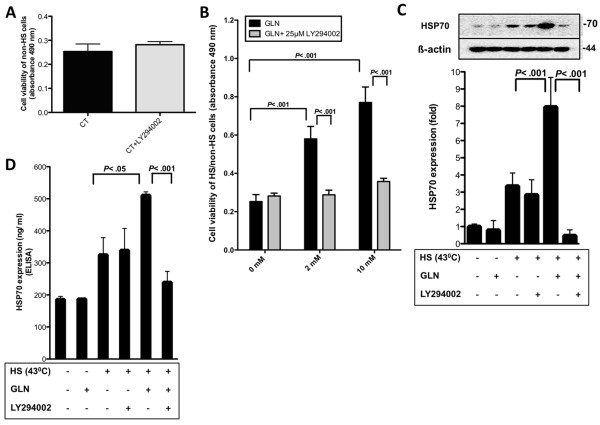
**GLN is protective via PI3-K/Akt-Hsp70 signaling after HS. A**) IEC-6 cells were treated with/without 1 h prior LY294002 (25 μM) treatment under non-stressed conditions (37°C). Cell viability was measured via MTS assay. Results are shown as mean ± SEM (n = 4). **B**) Cells were treated for 1 h with or without LY294002 (25 μM) before they were treated with 0, 2, or 10 mM GLN. Cell survival, following lethal HS (44°C), was measured via MTS assay. All groups were normalized to their non-HS controls to account for differences in cell growth. Assays were carried out in triplicate, experiments were performed 4 times and are shown as mean ± SEM. **C**) IEC-6 cells were treated with 0 mM or 10 mM GLN with or without LY294002 (25 μM) 1 h prior treatment under basal and stressed conditions (43°C). Hsp70 expression was determined by Western blot analysis (3 h recovery). In addition, ß-actin was measured to normalize total blotted protein. Data are shown as mean fold change relative to 0 mM GLN ± SEM (n = 4). **D**) Cells were treated as described in Figure 1C. Hsp70 expression is shown via ELISA in ng/ml ± SEM (n = 4).

The PI3-K/Akt pathway is an intracellular signaling pathway essential in apoptosis [25]. Our laboratory has shown, that GLN’s cytoprotective effect is, at least in part, mediated by increased Hsp70 expression [2]. In this study, we investigated cell viability in conjunction with PI3-K inhibitor LY294002 (25 μM) and GLN after thermal injury in IEC-6 cells and were interested whether Hsp70 expression is regulated via PI3-K/Akt signaling. MTS assays showed that GLN treatment increased cell survival in a dose-dependent manner in IEC-6 cells after lethal HS (*P* < .001) (Figure 1B). After demonstrating that 25 μM LY294002 is not toxic to IEC-6 cells (Figure 1A), we confirmed that PI3-K/Akt signaling was involved in GLN’s protective mechanism after HS as LY294002 (25 μM) attenuated GLN’s protection significantly (*P* < .001) (Figure 1B). This result confirms our previously published data that GLN + LY294002 (25 μM) treatment increased cleaved Caspase-3 and cleaved PARP levels in heat-stressed IEC-6 cells [28], suggesting the involvement of PI3-K/Akt signaling in GLN-protective mechanism in IEC-6 cells after thermal injury. To determine the effect of LY294002 (25 μM) on GLN-mediated Hsp70 expression, we examined Hsp70 levels after HS in IEC-6 via Western blotting and Hsp70 ELISA. Cells treated with 10 mM GLN showed increased Hsp70 levels after HS via Western blot (*P* < .001) (Figure 1C) and ELISA experiments (*P* < .05) (Figure 1D). IEC-6 cells treated with LY294002 (25 μM), however, showed a significant decrease in GLN-mediated Hsp70 levels in both, Western blots (*P* < .001) (Figure 1C) and Hsp70 ELISA experiments (*P* < .001) (Figure 1D).

### PI3-K/Akt signaling regulates FN expression and does not signal downstream of GLN-mediated FN-Integrin osmosignaling after HS

**Figure 2:**
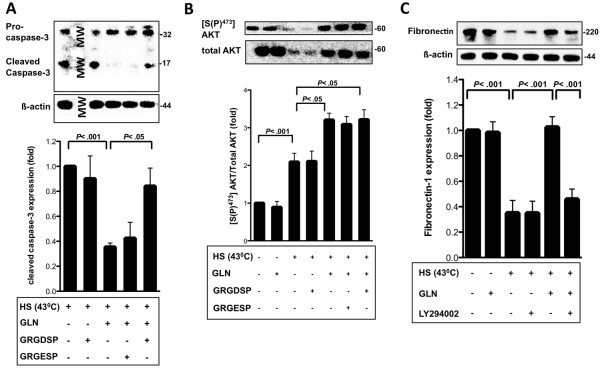
**PI3-K/Akt siganling regulates FN-expression and does not signal downstream of GLN-mediated FN-Integrin osmosignaling after HS. A**) IEC-6 cells were treated for 1 h with either media, GRGDSP (50 μM), or GRGESP (50 μM), prior to GLN treatment (0 mM or 10 mM) under basal (37°C) and stressed conditions (43°C). Procaspase-3, cleaved caspase-3 levels and ß-actin were measured via Western blot and cleaved Caspase-3 levels are presented as fold change ± SEM (n = 3). **B**) IEC-6 cells were treated as described in Figure 2A. Representative Western blots of 3 independent experiments of [S(P)^473^]Akt and total Akt are shown as mean fold change relative to total Akt ± SEM and normalized to 0 mM GLN. **C**) FN levels with/without GLN (10 mM) and LY294002 (25 μM) treatment under unstressed (37°C) or stressed conditions (43°C) were determined by Western blot after 3 h recovery. Densitometric analysis of FN expression as mean fold change relative to 0 mM GLN cells ± SEM (n = 3-8).

FN-Integrin signaling is essential in GLN-mediated protection in IEC-6 cells after HS [24]. In this study, we were able to confirm these results: By using the FN-Integrin inhibitor GRGDSP (50 μM), we demonstrated that GLN-mediated decreases in cleaved Caspase-3 levels increased after FN-Integrin interaction inhibitor GRGDSP treatment (*P* < .05). Its inactive control peptide GRGESP (50 μM) had no effect (Figure 2A). Next, we investigated whether FN-Integrin signaling is essential in GLN-mediated Akt phosphorylation, since it has been documented that Akt is essential for the “inside-out” activation of integrins, which in turn mediates matrix assembly (e.g. FN) in fibroblasts [21]. Therefore, we investigated the role of GLN-mediated PI3-K/Akt signaling in conjunction with FN-Integrin signaling. HS decreased total Akt levels, however, when phosphorylated Akt levels were normalized to their total Akt levels, HS increased phorphorylated Akt by 2-fold and addition of 10 mM GLN enhanced this effect by 3-fold, as demonstrated in our previous study [28]. However, adding GRGDSP (50 μM) and control GRGESP (50 μM) to the GLN-treated group did not change the 3-fold increases in GLN-mediated Akt phosphorylation (*P* < .05) (Figure 2B), suggesting that PI3-K/Akt signals before or in parallel of FN-Integrin signaling.

FN expression is important to regulate cell survival and to interact with integrins for osmosignaling [32]. Degradation of FN leads to less interaction with integrins, reducing osmosignaling. Our results confirmed that HS decreased FN (*P* < .001). However, GLN inhibited this decrease after HS, as demonstrated by our laboratory [24]. In this experiment, we added PI3-K/Akt inhibitor LY294002 (25 μM) to GLN-treated groups and demonstrated that GLN was not able to prevent FN expression after HS when LY294002 (25 μM) was added (*P* < .001) (Figure 2C).

### GLN is protective by dephosphorylating p38MAPK downstream of GLN-mediatedFN-Integrin osmosignaling after HS

**Figure 3:**
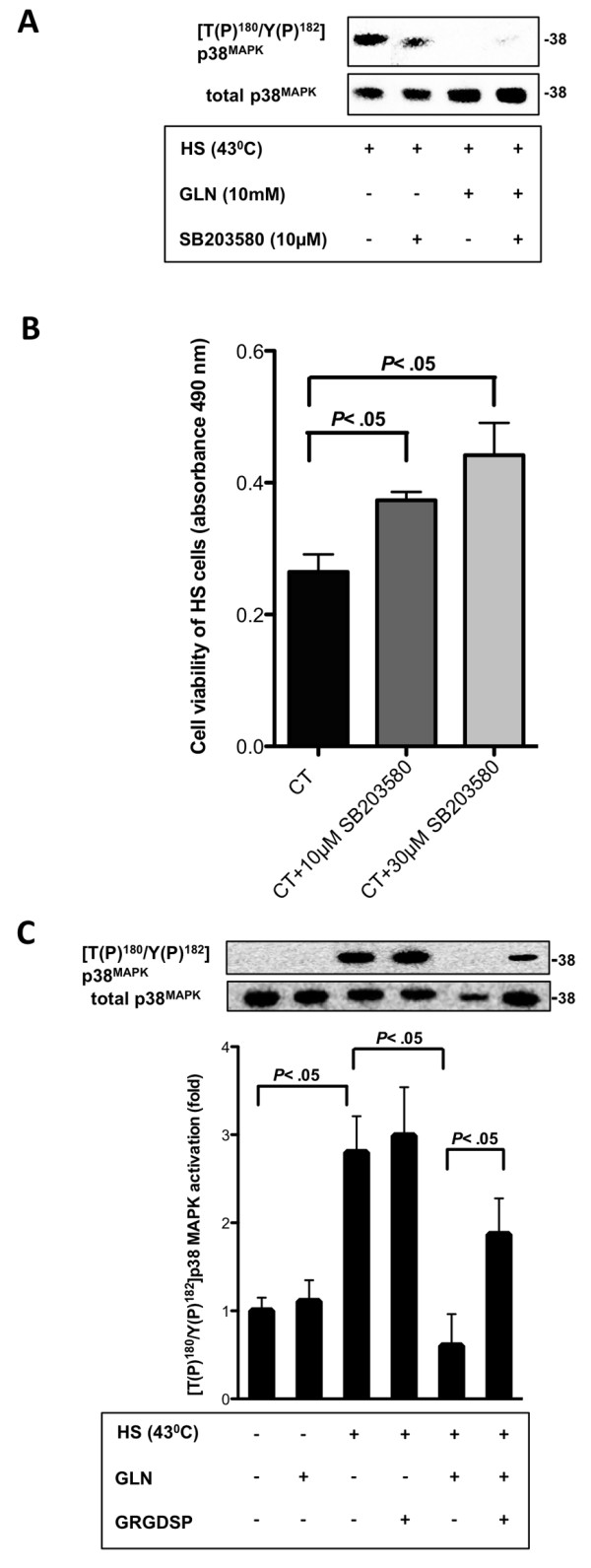
**GLN is protective by dephosphorylating p38MAPK downstream of GLN-mediated FN-Integrin osmosignaling after HS. A**) Cells were treated with 0 mM or 10 mM GLN with or without SB203580 (10 μM) 1 h prior treatment under stressed conditions (43°C). A representative Western blot of [T(P)^180^/Y(P)^182^]p38MAPK and total p38MAPK levels is shown. **B**) IEC-6 cells were treated with/without 1 h prior SB203580 (10 μM and 30 μM) treatment under stressed conditions (44°C). Cell survival was measured via MTS assay. Results are shown as mean ± SEM (n = 4). **C**) IEC-6 cells were treated as described in Figure 2A. Western blots of [T(P)^180^/Y(P)^182^]p38MAPK and total p38MAPK levels are shown. P38MAPK phosphorylation is revealed as mean fold change relative to total p38MAPK ± SEM and ratioed to 0 mM GLN (n = 4).

Since p38MAPK’s pro- or anti-apoptotic functions appear to be dependent on the cell type and cellular content [33,34], we examined its role in GLN’s protective mechanism in IEC-6 cells after hyperthermia. Therefore, we used the p38MAPK phosphorylation inhibitor SB203580. We confirmed via Western blot that SB203580 (10 μM) was able to attenuate p38MAPK phosphorylation (Figure 3A). After demonstrating in our recent publication that SB203580 (10 μM) increased cell survival after HS in IEC-6 cells, we were interested as whether higher concentrations of SB203580 would further increase cell viabilty [28]. Herein, we were able to show that SB203580 (10 μM and 30 μM) was able to increase cell survival after lethal HS in a dose-dependent manner (Figure 3B), suggesting that dephosphorylation of p38MAPK correlates with cell survival. Next, we investigated whether FN-Integrin signaling is essential in GLN-mediated p38MAPK dephosphorylation. Figure 3C confirmed as previously published [28] that HS increased p38MAPK phosphorylation (*P* < .05) and 10 mM GLN attenuated its phosphorylation significantly (*P* < .05). GRGDSP (50 μM) altered GLN-mediated p38MAPK phosphorylation (*P* < .05), indicating involvement of FN-Integrin signaling in GLN-mediated p38MAPK signaling (Figure 3C).

## Discussion

Critical illness and inflammatory injuries, such as sepsis, shock, and inflammatory bowel disease, are one of the leading causes of morbidity and mortality in the US and around the world [35,36]. At present, studies to define new therapeutic interventions that could protect tissues and cells against injury, attenuate inflammation, and preserve metabolic function are fields of intense investigation. GLN seems to be a potential therapeutic in intestinal diseases, however currently, the molecular mechanisms and the initiation steps involved in GLN-mediated protection are not well understood.

Our study provides new mechanistic insights into GLN’s initial anti-apoptotic steps in the gut after thermal injury. In this study, we show that FN-Integrin, p38MAPK, and PI3-K/Akt signaling play essential roles in GLN-mediated cell survival signaling. GLN activated PI3-K/Akt signaling independently from FN-Integrin signaling after HS (Figure 2B), prevented FN expression (Figure 2C), and increased HSP70 expression (Figure 1C and D) to prevent apoptosis (Figure 1B). Akt1 is crucial for the “inside-out” activation of integrins, which in turn mediates matrix assembly [21] and is involved in the activation of integrins, which is an essential key step necessary for adhesion in endothelial cells, regulating ECM assembly [21,37]. Thus, it seemed reasonable to hypothesize that PI3-K/Akt signaling could regulate GLN-mediated FN expression and FN-Integrin signaling via “inside-out” signaling in intestinal epithelial cells to prevent cell death after intestinal injury.

In this study, we show for the first time that PI3-K/Akt signaling regulates GLN-mediated FN expression after hyperthermia (Figure 2C). FN expression is important for cell survival [32] and is essential in GLN’s protective mechanism [24]. Degradation of FN leads to less interaction with integrins, which reduces osmosignaling. Here, evidence is presented that PI3-K/Akt signaling regulates GLN-mediated FN expression after HS, possibly via “inside-out” signaling to activate FN-Integrin interactions. Our laboratory recently published that FN-Integrin interaction inhibitor GRGDSP and the ERK1/2 kinase inhibitor PD98059 attenuated GLN-mediated increases in Hsp70 expression [24]. Thus, it was important to determine if the PI3-K-inhibitor LY294002 was also able to decrease GLN-mediated Hsp70 expression. The results in Figure 1C and D indicated that PI3-K/Akt signaling regulated GLN-mediated Hsp70 enhancement after HS. If our hypothesis is correct and PI3-K/Akt signaling is able to regulate both FN expression and the activation of FN-Integrin signaling as part of GLN’s protective effects, this would be in agreement with our results showing that both GRGDSP and LY294002 decrease GLN-mediated Hsp70 expression. However, it could also be possible that PI3-K/Akt signals in parallel to FN-Integrin signaling to prevent apoptosis via increased Hsp70 expression. This key area of research still needs to be investigated in future studies. Future experiments with LY294002 or PI3-K siRNA in conjunction with ERK1/2 and p38MAPK activation should yield important new mechanistic insights.

As ERK1/2 activation was involved in GLN-mediated FN-Integrin signaling and as Sakiyama et al. previously reported that GLN regulated cell survival by p38MAPK pathway that affected autophagy [38], we evaluated if GLN-mediated p38MAPK dephosphorylation was also regulated via FN-Integrin signaling. Exposure of intestinal epithelial cells to the FN-Integrin inhibitor GRGDSP showed that p38MAPK serves as downstream mediator of GLN-mediated FN-Integrin signaling (Figure 3C). What role GLN-mediated FN-Integrin-p38MAPK signaling plays related to autophagy will be an interesting field of research in future studies.

In conclusion, as was found for ERK1/2, p38MAPK is regulated via GLN-mediated FN-Integrin signaling in intestinal epithelial cells after thermal injury. We assume that the enhancement of HSP by HS may be the secondary change for protection against the activation of p38MAPK. However, the specific order of interactions between PI3-K/Akt, HSP, and p38MAPK are not known at present.

**Figure 4:**
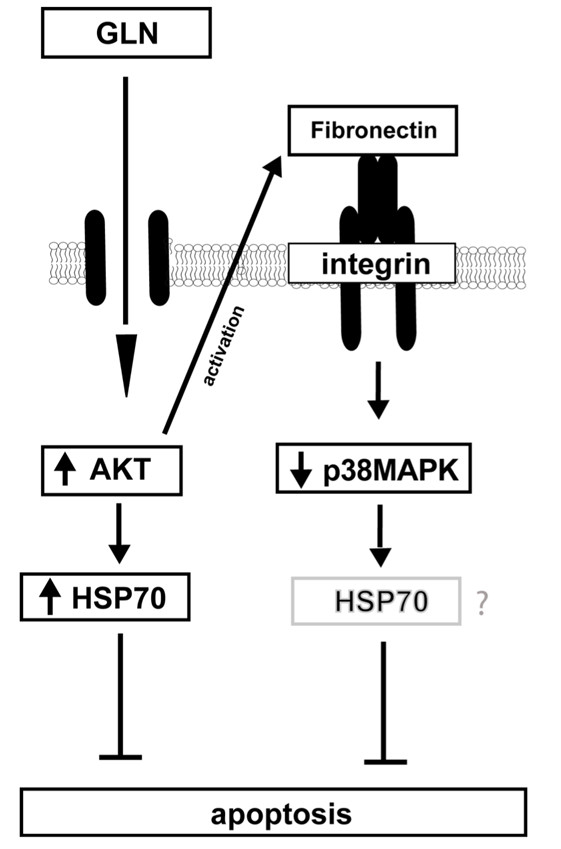
**Proposed working model.** GLN is protective in IEC-6 cells by activating PI3-K signaling which prevents FN expression and increases HSP70 expression after thermal injury. Furthermore, GLN is protective by dephosphorylating p38MAPK via GLN-mediated FN-Integrin signaling after HS. GLN-mediated PI3-K/Akt signaling, however, seems to be independent of FN-Integrin signaling.

GLN-mediated PI3-K signaling, however, either happens before FN-Integrin signaling or simultaneously after injury in the intestine. We suggest that GLN-mediated PI3-K/Akt signaling regulates FN expression and possibly FN-Integrin osmosignaling after injury. This induces Hsp70 expression, which is known to prevent apoptosis. Figure 4 shows an overview of our current working hypothesis for GLN’s cellular anti-apoptotic effect. “The effect of p38MAPK inhibition on HSP70 expression will need to be evaluated in future studies. We hypothesize that inhibition of p38MAPK by GLN may have minimal effects on GLN-mediated increases in HSP70 expression since we were able to show in our previous publication that SB203580 did not increase GLN’s beneficial effect on cell viability in MTS cell survival assays [28]. However, SB203580 increased cell survival in heat stressed groups in a dose dependent manner (Figure 3B)”.

Continued basic and clinical research considering GLN as a potential therapeutic agent in gastrointestinal disease is essential, since GLN has dynamic effects on the gastrointestinal tract and remains an extremely promising nutrient for metabolic support of patients with intestinal disorders. This work gives new and potentially clinically relevant mechanistic insights into GLN-mediated molecular cell survival pathways. These results warrant clinical translation to assess if clinical outcome of clinical states of gut injury can be improved by GLN treatment and/or by targeting the molecular pathways found in this study.

## Abbreviations

GLN: Glutamine; HS: Heat stress; HSP: Heat shock protein; FN: Fibronectin; PI3-K: Phosphoinositol 3-kinase; FBS: Fetal bovine serum; IEC-6 cells: Intestinal epithelial-6 cells; ECM: Extracellular matrix protein.

## Competing interests

The author’s declare that they have no competing interests.

## Authors’ contributions

SN: study concept and design; acquisition of data; analysis and interpretation of data; statistical analysis; drafting the manuscript. CB acquisition of data. PW study concept and design, obtained funding, technical and material support, study supervision, critical revision of the manuscript for important intellectual content. All authors read and approved the final manuscript.
